# Effects on management and outcome of severe sepsis and septic shock patients admitted to the intensive care unit after implementation of a sepsis program: a pilot study

**DOI:** 10.1186/cc8029

**Published:** 2009-09-03

**Authors:** Massimo Girardis, Laura Rinaldi, Lara Donno, Marco Marietta, Mauro Codeluppi, Patrizia Marchegiano, Claudia Venturelli, the 'Sopravvivere alla Sepsi 'group of the Modena-University Hospital

**Affiliations:** 1 Department of Anaesthesiology and Intensive Care, University of Modena and Reggio Emilia and University Hospital of Modena; L.go del Pozzo, Modena, 41100, ITALY; 2 Department of Haematology, University Hospital of Modena; L.go del Pozzo, Modena, 41100, ITALY; 3 Department of Infectious Diseases, University Hospital of Modena; L.go del Pozzo, Modena, 41100, ITALY; 4 Medical Direction, University Hospital of Modena; L.go del Pozzo, Modena, 41100, ITALY; 5 Microbiology and Virology Unit, University Hospital of Modena; L.go del Pozzo, Modena, 41100, ITALY

## Abstract

**Introduction:**

The application in clinical practice of evidence-based guidelines for the management of patients with severe sepsis/septic shock is still poor in the emergency department, while little data are available for patients admitted to the intensive care unit (ICU). The aim of this study was to evaluate the effect of an in-hospital sepsis program on the adherence to evidence-based guidelines and outcome of patients with severe sepsis/septic shock admitted to the ICU.

**Methods:**

This prospective observational cohort study included 67 patients with severe sepsis/septic shock admitted to a multidisciplinary ICU at a University Hospital from January 2005 to June 2007. Compliance to 5 resuscitation and 4 management sepsis interventions and in-hospital mortality were measured following an educational program on sepsis for physician and nurses of all hospital departments and hospital implementation of a specific protocol for recognition and management of patients with severe sepsis/septic shock, including an early consultation by a skilled 'sepsis team'.

**Results:**

During the study period, the compliance to all 9 interventions increased from 8% to 35% of the patients (*P *< 0.01). The implementation of resuscitation and management interventions was associated with a lower risk of in-hospital mortality (23% vs 68% and 27% vs 68%, *P *< 0.01). In the latter 2 semesters, after activation of the 'sepsis team', in-hospital mortality of ICU septic shock patients decreased by about 40% compared with the previous period (32% vs 79%, *P *< 0.01).

**Conclusions:**

In our experience, an in-hospital sepsis program, including education of health-care personnel and process-changes, improved the adherence to guidelines and the survival rate of patients with severe sepsis/septic shock admitted to the ICU.

## Introduction

The high incidence, costs and mortality rate of patients with sepsis in the recent years has led the critical care scientific community to develop specific strategies aimed to improve the outcome of these patients [1-4]. In 2004, the Surviving Sepsis Campaign (SSC) guidelines [3] recommended a series of diagnostic and therapeutic interventions whose implementation was expected to lead to a survival benefit in patients with severe sepsis/septic shock. Afterwards, to facilitate the application of these guidelines in clinical practice, the Institute for Healthcare Improvement (IHI) proposed the severe sepsis resuscitation (6-hours) and management (24-hours) bundles, that integrate the interventions described above. Nevertheless, the application of these bundles so far has been demonstrated to be quite poor in most surveys, confirming the difficulty of transferring evidence to the clinical practice [4-12].

The main purpose of our study was to evaluate the effects of a 'surviving sepsis' in-hospital project, including specific educational program and operative protocols, on the adherence to evidence-based guidelines. Moreover, we sought to assess if such a project could improve the outcome of patients with severe sepsis/septic shock admitted to an intensive care unit (ICU).

## Materials and methods

### Design, setting and population

This prospective observational study enrolled consecutive patients with a diagnosis of severe sepsis/septic shock admitted to an ICU of the 780-bed University Hospital of Modena from January 2005 to June 2007. The study was approved by the local ethical committee and the need for informed consent was waived in view of the observational and anonymous nature of the study. The ICU consists of nine beds and approximately 800 adult patients are admitted annually (70% surgical patients). Staffing at any time consists of one attending physician, one resident physician and three to four nurses.

The inclusion criteria were: a) documented or suspected infection; b) two or more systemic inflammatory response syndrome criteria [13] and c) the onset of an organ dysfunction related to infection: gas exchange impairment (partial pressure of arterial oxygen (PaO_2_)/fraction of inspired oxygen (FiO_2_) < 250 mmHg), mean arterial pressure (MAP) below 65 mmHg, acute renal dysfunction (1.5-fold baseline creatinine increase or urine output < 0.5 ml/Kg/h for two hours), total bilirubin above 4 mg/dL, platelet count below 80,000 cells/mm^3 ^(or a 100,000 cells/mm^3 ^decrease) or lactate blood concentration above 4.0 mM. Patients with persistence of MAP below 65 mmHg after an adequate fluid infusion (see below) were classified as having septic shock. Patients with severe decompensated chronic liver disease included in the waiting list for liver transplantation were excluded from the study.

### Data collection

Data collection began one month after the start of an in-hospital educational program on sepsis (see below) and only the first episode of severe sepsis/septic shock was considered in each patient. The management of patients was evaluated by analysis of interventions and sepsis bundles [3]. We identified five resuscitation (6-hours bundle) and four management (24-hours bundle) interventions: blood cultures collection before antibiotic administration; empiric antibiotic therapy within three hours from diagnosis; control of infection source within six hours; adequate fluid resuscitation before vasopressor administration; central venous oxygen saturation (ScvO_2_) above 70% within six hours; blood glucose median below 150 mg/dL in the first 24 hours; low-dose hydrocortisone administration in association with vasopressor support; recombinant human activated protein C (rhAPC) if administration indicated; plateau inspiratory pressure below 30 cmH_2_O in patients with acute lung injury (ALI)/adult respiratory distress syndrome (ARDS). The term adequate fluid resuscitation indicates a central venous pressure above 6 mmHg (above 8 mmHg if mechanically ventilated) or a global end-diastolic volume by trans-pulmonary thermodilution (PiCCO system, Pulsion, Germany) above 700 ml/m^2^.

Two of the authors (LR and LD) not involved in the clinical management of the patients, collected the above interventions by analysis of clinical charts and any uncertain data was audit with the attending physician. The interventions were classified as completed and not completed. An intervention not applied because not applicable (e.g. low plateau inspiratory pressure in patient without ALI/ARDS) was defined as completed. The time zero for bundles timing was the time in which the three study inclusion criteria were documented by clinical notes. Type of admission, grade of sepsis, primary site of infection, simplified acute physiology score (SAPS) II and simplified organ failure assessment (SOFA) score the day of sepsis diagnosis [14,15], ICU and hospital length of stay, and hospital mortality were also recorded for each patient. Predicted hospital mortality was calculated by SAPS II score.

### Hospital program

The education phase of our hospital program named "Sopravvivere alla Sepsi nel Policlinico di Modena" (Surviving to Sepsis in Policlinico Hospital of Modena) started on November 2004 and continued throughout the study period. It included basic, advanced and refresh courses with conference lectures and practice training for nurses and physicians of all hospital departments. From November 2004 to June 2007 almost 250 physicians (out of 400) and 300 nurses (out of 950) of our hospital participated in educational courses. A specific protocol for early recognition and management of patients with severe sepsis/septic shock was prepared, approved and promoted (e.g. specific meetings, hospital intra-net, poster displayed in the staff working area) in all hospital wards (June 2006). The protocol includes: i) clinical data needed for severe sepsis/septic shock identification; ii) instruction for 'sepsis team' activation; iii) detailed instructions for early goal directed resuscitation, collection of microbiological samples and antibiotic therapy; and iv) special recommendations on bicarbonate use, low-dose dopamine and glycaemia control. The sepsis team is available 24 hours per day and is formed by two attending physicians: an intensivist and an infectious disease specialist. The team is activated by and collaborates with the attending physician and the nursing department staff in providing the interventions required for each patient with severe sepsis and septic shock (e.g. placing central venous line, measuring central venous pressure, providing non-invasive ventilation, assessing for antibiotic strategy and other specific therapy). After the activation by a dedicated telephone number, the time period for team sepsis consultation should be shorter than 60 minutes in patients with severe sepsis and 30 minutes in patients with septic shock. The sepsis team activity (e.g. frequency and percentage of appropriate activation, mean time before consultation, percentage of ICU admission, patient outcome) is regularly recorded and discussed with members of the "Sopravvivere alla Sepsi" group and with the hospital administrators.

### Statistical analysis

The outcome measurements included intervention compliance, ICU and in-hospital length of stay and in-hospital mortality. For data analysis, the study period was divided: in semesters, in order to assess the progression of learning process and in two periods, before and after June 2006, in order to assess the impact of 'sepsis team' on patient outcome. Students' t-test, chi-squared, Fisher's exact test, and analysis of variance single-factor analysis were used when appropriate. Univariate and multivariate logistic regression were performed, with hospital mortality as dependent variable and individual interventions, bundles and sepsis team admission as independent variables. Variables with *P *< 0.20 from univariate analysis were included in the backward logistic regression model that was also corrected for possible confounders such as age, SOFA and SAPS II scores, the presence of shock, lactate blood concentration (first data after study inclusion) and sepsis team period. The goodness of fit was assessed by the Hosmer-Lemeshow test. A value of *P *< 0.05 was considered significant. The statistical software package SPSS 15.0 (SPSS Inc., Chicago, IL, USA) was used for statistical analysis.

## Results

From January 2005 to June 2007, 87 patients met criteria for study inclusion, but 20 patients were excluded because they were affected by chronic decompensated cirrhosis and were on the waiting list for liver transplantation. Comparing the five semesters of the study period, no differences were observed in the number of patients, age, gender, type of admission (i.e. surgical and emergency department), primary site of infection, SAPS II and hospital length of stay. Percentage of septic shock patients, SOFA score, ICU length of stay and in-hospital mortality decreased (*P *> 0.05) during the study period (Table 1).

**Table 1:** Number, age, sex, primary site of infection, grade of sepsis, severity scores, length of stay and mortality of patients subdivided for semesters

Parameters	Total	January to June 2005	July to December 2005	January to June 2006	July to December 2006	January to June 2007
Patients (n)	67	13	11	10	13	20
Age (years; mean ± SD)	63 ± 16	65 ± 9	69 ± 13	66 ± 18	58 ± 17	61 ± 20
Female (n, %)	23(46)	1 (8)	3 (27)	4 (40)	6 (46)	9 (45)
ED admissions (n, %)	16 (24)	1 (8)	2 (18)	3 (30)	4 (31)	6 (30)
Surgical admissions (n, %)	38 (56)	8 (61)	8 (73)	4 (40)	7 (54)	11 (55)
Primary site of infection						
*Pneumonia(%)*	36	38	36	40	31	35
*Intra-abdominal (%)*	27	15	18	40	38	25
*Blood (%)*	15	15	27	0	15	15
*Urinary tract(%)*	10	8	9	10	8	15
*Surgical wound(%)*	5	8	0	0	8	5
*Other (%)*	7	15	9	10	0	5
Septic shock (n, %)	50 (75)	11 (85)	10 (91)	7 (70)	9 (69)	13 (65)
Blood lactate > 4 mmol/L (n, %)	28 (42)	4 (31)	8 (73)	3 (30)	6 (46)	7 (35)
SAPS (mean ± SD)	53 ± 21	50 ± 15	53 ± 29	61 ± 24	47 ± 19	55 ± 21
SOFA (mean ± SD)	9.7 ± 3.9	12.3 ± 4.0	10.1 ± 4.6	10.1 ± 4.0	8.4 ± 3.4	8.4 ± 2.9
ICU LOS (days; mean ± SD)	16 ± 19	24 ± 33	24 ± 10	16 ± 24	16 ± 17	14 ± 9
H LOS (days; mean ± SD)	44 ± 38	53 ± 34	31 ± 38	38 ± 49	56 ± 42	42 ± 25
H mortality overall (n, %)	33 (49)	9 (69)	7 (64)	7 (70)	3 (23)	7 (35)
H mortality septic shock (n, %)	30 (60)	9 (82)	8 (80)	6 (86)	2 (22)	5 (38)

ED = emergency department; ICU = intensive care unit; H = hospital; LOS = length of stay; SAPS = simplified acute physiology score; SD = standard deviation; SOFA = simplified organ failure assessment.

**Figure 1:**
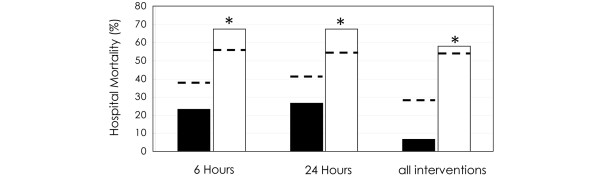
Mortality of patients with (black column) and without (white column) implementation of 6-hours bundle, 24-hours bundle and all interventions.  For each group of patients the predicted mortality by simplified acute physiology score (SAPS) II is also reported (dotted line). * *P *< 0.05 comparing patients with and without bundles compliance.

The interventions compliance increased (*P *< 0.05) from January 2005 to June 2007 for all but the glycaemia control and adequate fluid resuscitation. In the same way, the compliance with 6-hour resuscitation and 24-hour management bundles as well as with all interventions increased (*P *< 0.01) (Table 2). The implementation of bundles was associated (*P *< 0.01) with a decrease of in-hospital mortality (Figure 1). The characteristics of patients with and without all interventions compliance were similar, except for age (55 ± 12 vs 65 ± 13 years), sex (60 vs 27% female) and SAPS II (44 ± 13 vs 56 ± 21; *P *< 0.05). Nevertheless, the differences between observed mortalities and expected mortalities by SAPS II were favourable (*P *< 0.05) in patients with bundles and all interventions compliance (Figure 1).

**Table 2:** Percentage of patients with completion of interventions and bundles subdivided for semesters of analysis

Intervention	Total	January to June 2005	July to December 2005	January to June 2006	July to December 2006	January to June 2007
Blood cultures collection*	83	77	73	80	92	95
Antibiotic therapy (3 hours)*	95	92	82	100	100	100
Infection source control* ^§^	86	85	82	70	92	100
Adequate fluid resuscitation	98	92	100	100	100	95
ScvO_2 _optimization*	61	46	45	50	92	70
Glycaemia control	93	92	100	100	92	80
Low-dose hydrocortisone*	73	31	82	80	85	90
rhAPC*	66	54	45	70	77	85
PiP < 30 cmH_2_O*	79	46	82	80	85	100
6-hours bundle	45	38	9	20	77	60
24-hours bundle	45	8	36	50	62	60
All interventions	22	8	0	10	46	35
Sepsis team admissions*	33	0	0	0	85	55

Data are expressed as percentage of patients. * *P *< 0.05 comparing the semesters; ^§ ^Source control details: 38 surgical patients: 21 control by surgery, 3 radiological drainage, 8 control not necessary, 6 control not achieved within 6 hours. 29 medical patients: 6 radiological drainage, 6 central venous line removal, 13 control not necessary, 4 control not achieved within 6 hours.

PiP = plateau inspiratory pressure; rhAPC = recombinant human activated C protein; ScvO_2 _= central venous oxygen saturation.

**Figure 2:**
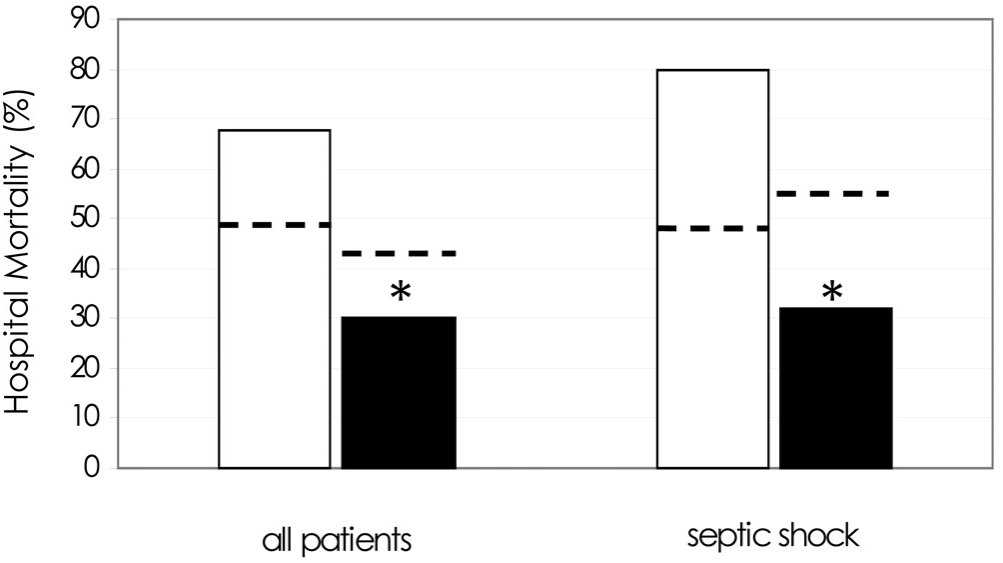
In-hospital mortality before (white columns) and after (black columns) 'sepsis team' activation (June 2006) in all population and in septic shock patients.  For each group of patients, the predicted mortality by simplified acute physiology score (SAPS) II is also reported (dotted line). * *P *< 0.05 before and after sepsis team activation.

In-hospital mortality decreased by about 40% (*P *< 0.01) during the past two semesters (i.e. after 'sepsis team' activation, July 2006 to June 2007) compared with the previous ones (January 2005 to June 2006; Figure 2). Patients of these two study periods were similar in age, type of admission, primary site of infection and SAPS II, but in the two latter semesters SOFA score (8.4 ± 3.1) and percentage of septic shock patients (66%) were lower (*P *< 0.05) than in the earlier three semesters (10.9 ± 4.2 and 82%). Considering only septic shock patients in the two study periods, no differences were observed in demographic characteristics whereas the in-hospital mortality decreased (*P *< 0.01) in the two latter semesters (Figure 2).

The univariate logistic regression showed that odds ratio (OR) for in-hospital mortality was reduced (*P *< 0.05) by compliance to infection source control, ScvO_2 _optimisation, rhAPC administration, 6-hours and 24-hours bundles, all interventions together and team sepsis. Multivariate logistic analysis with adjustment for possible confounders indicated that 6-hours bundle implementation as well as 24-hours bundle were independently (*P *< 0.05) associated with lower in-hospital mortality (Table 3).

**Table 3:** Univariate and multivariate logistic analysis for in-hospital mortality

	Odds ratio	95% confidence interval	*P *value
**Univariate analysis**			

Infection source control	0.12	0.02 to 0.89	0.031

ScvO_2 _optimization	0.30	0.10 to 0.83	0.025

rhAPC	0.18	0.06 to 0.58	0.004

6-hours bundle	0.17	0.06 to 0.50	< 0.001

24-hours bundle	0.19	0.05 to 0.65	0.004

All interventions	0.05	0.01 to 0.31	< 0.005

Team sepsis activation	0.28	0.10 to 0.79	0.015

**Multivariate analysis**			

6-hours bundle	0.15	0.03 to 0.63	0.010

24-hours bundle	0.12	0.02 to 0.52	0.005

Hosmer-Lemeshow test: *P *= 0.819.

rhAPC = recombinant human activated C protein; ScvO_2 _= central venous oxygen saturation.

## Discussion

The main findings of our study were that an in-hospital program dedicated to sepsis, including health-care personnel education and specific process changes, improved not only the adherence to evidence-based guidelines in clinical practice, but also the survival rate of patients with severe sepsis and septic shock admitted to the ICU. Also, the adherence to international guidelines provided more appropriate blood cultures, optimization of SvcO_2 _and adherence to indications for rhAPC, steroids and protective ventilation.

In accordance with the indications of IHI for the local implementation of the SSC, a few months after the publication of the international guidelines [3] our hospital program started with an educational phase. It involved a large number of physicians and nurses, particularly from those wards implicated in the management of patients with severe sepsis/septic shock. The early establishment of a working group on sepsis, including reference nurses and physicians from all the hospital departments, was a key point in motivating the department staff to an active collaboration. Nevertheless, the high turn-over of residents and nurses led to a progressive impoverishment of skilled personnel. To overcome this problem, since 2006 a continuous educational program has been planned as a form of required education for health-care personnel at the hospital.

The compliance to evidence-based interventions at the beginning of the hospital program was very similar to that reported by others in emergency departments (ED) [9-11]. Unfortunately, so far, few data have been reported on the implementation of sepsis bundles in ICU. Ferrer and colleagues [12] recently reported a very low compliance to resuscitation (5.3%) as well as management (10.9%) bundles before an education program in Spanish ICUs. On the other hand, Gao and colleagues [8] observed in ICU patients a rate of satisfaction of 6-hours sepsis bundles (59%) higher than that observed in our study. However, in the study by Gao and colleagues the 6-hours resuscitation bundles did not include the assessment and optimization of ScvO_2_, that is the intervention was more frequently uncompleted in our patients as well as in other studies [9,11,12].

The compliance to evidence-based guidelines increased during the study period and led mainly to an increase of blood culture collection before antibiotic therapy, optimization of ScvO_2_, steroid use in shocked patients, adherence to indications for rhAPC and protective ventilation. Indeed, adherence to glycaemia control in our experience slightly decreased during the study period probably because of a great concern of the ICU staff for hypoglycemia-related complications originated by preliminary results of clinical trials [16].

In the latter two semesters, the adherence to 6-hours resuscitation bundles suddenly improved (Table 1). This can be attributed to the activation of process changes in the hospital management of patients with sepsis that provided an early identification and appropriate treatment of patients with organ dysfunction both before and after ICU admission. Nevertheless, also in the last period of the study we were able to complete all the sepsis bundles only in 35 to 40% of the patients. Numerous activities, besides continuous educational programs, have been put in action to further improve this result: departmental audit on specific sepsis cases, procalcitonin measurement 24 hours per day and a sepsis dedicated laboratory panel including lactate and the parameters needed for organ dysfunction assessment.

Many studies have indicated that the implementation of interventions recommended by evidence-based guidelines are associated with outcome benefits in severe sepsis patients [5-10,12]. However, the majority of these studies were carried out in EDs including out-of-hospital patients with community acquired infection. Very few data are available about the effectiveness of this strategy in ICU patients with different provenance (i.e. ED, surgical or medical wards) and type of infection (i.e. community or hospital acquired) [7,8,12]. Our data also indicated that in such a setting the compliance to evidence-based interventions improve the outcome of patients with severe sepsis/septic shock. Furthermore, the multivariate analysis including a correction for SAPS II and SOFA scor-, showed that the complete adherence to 6 hours and 24-hours interventions is associated with a significant OR reduction for in-hospital mortality.

As far as single interventions are concerned, the association between ScvO_2 _of 70% or more and improved outcome in patients with severe sepsis/septic shock has been widely demonstrated in EDs [5,10,17], but this is the first time that the same figure is reported in ICU patients. Van Beest and colleagues [18] recently reported that the incidence of low ScvO_2 _in acutely admitted septic shock is very low in Dutch ICUs. In our centre, despite changes in management processes, the incidence of patients with low or unknown ScvO_2 _within six hours from severe sepsis diagnosis was still around 20% in the past year. Risks and benefits of rhAPC in patients with severe sepsis/septic shock have been largely discussed and a further discussion on this issue is certainly beyond the aims of this paper. However, we observed that the adherence to the SSC guidelines [3] for the use of rhAPC was associated with a significant decrease in mortality. However, it must be underlined that the number of patients was low and that in the multivariate analysis none of the single interventions was associated with a significant change in OR for patient mortality.

As discussed above, the institution of a specific team for early sepsis management led to a significant improvement in outcome. This improvement regarded also the septic shock patients, already referred to the ICU before sepsis team institution. One can argue that the improvement could be due to an increased adherence to 24-hours bundle. However, after the sepsis team institution we observed a more remarkable improvement in 6-hours bundle. This suggests that the adopted process changes facilitated a quicker management of shocked patients.

Our study has some limitations. First, the study design (non-randomized) and the low number of patients involved so far do not allow us to draw any firm conclusions on the effect of single interventions, bundles and process change on sepsis outcome. Second, it has to be considered that the sepsis management model provided and analyzed in our study was according to the 2003 version of the SSC guidelines [4] and, therefore, is in some aspects different to that proposed by the more recent ones [19]. Third, as sepsis team institution and increased bundles compliance occurred simultaneously, we are not able to differentiate the actual role of one in respect to the other on the mortality reduction observed in the past year.

## Conclusions

In conclusion, our single-centre experience demonstrated the importance of specific program addressed to whole hospital departments for improving evidence-based practice and survival rate of patients with severe sepsis/septic shock admitted in ICU. In our model, a multidisciplinary approach and a specific team played a key role for education and for providing an early and appropriate sepsis management. A large number of patients and a more detailed assessment of sepsis team activity before ICU admission appears mandatory for a better understanding of this relevant issue.

## Key messages

• The application in clinical practice of evidence-based guidelines for management of patients with severe sepsis/septic shock is still unsatisfactory.

• An educational program directed to all hospital departments and specific in-hospital process changes for early patient management increased the compliance to sepsis guidelines and led to 45% absolute risk reduction for in-hospital death in patients with septic shock.

• The institution of a specific sepsis team seems to be a key point for providing the adequate management of in-hospital patient with severe sepsis/septic shock.

## Abbreviations

ALI: acute lung injury; ARDS: Adult respiratory distress syndrome; ED: emergency department; FiO_2_: fraction of inspired oxygen; ICU: intensive care unit; IHI: institute for healthcare improvement; MAP: mean arterial pressure; OR: odds ratio; PaO_2_: partial pressure of arterial oxygen; rhAPC: recombinant human activated protein C; SAPS: simplified acute physiology score; ScvO_2_: central venous oxygen saturation; SOFA: simplified organ failure assessment; SSC: Surviving Sepsis Campaign.

## Competing interests

MG has consulted for Eli-Lilly Italia; the remaining authors declare that they have no competing interests.

## Authors' contributions

MG has made substantial contributions to study conception and design, data analysis and has been involved in drafting the manuscript. LR has made substantial contributions to study conception and design, acquisition of data, statistical analysis and has been involved in drafting the manuscript. LD has made substantial contributions to study conception and design and acquisition of data. MM has made substantial contributions to study conception and design and has been involved in revising the manuscript for important intellectual content. MC has been involved in revising the manuscript for important intellectual content. PM has made substantial contributions to study conception and design. CV has made substantial contributions to study conception and design and data collection.

## Authors' information

*Members of the 'Sopravvivere alla Sepsi" group of the Modena University Hospital [20]: Baraghini F, Barbieri M, Bonucchi D, Borghi A, Cattani S, Cellini M, Corradi L, Donelli A, Fratti O, Guaraldi N, Leoni P, Lo Fiego E, Malagoli M, Moretti M, Petocchi B, Russo N, Serio L, Tazzioli G, Zito L.
